# *Paecilomyces variotii* as an Emergent Pathogenic Agent of Pneumonia

**DOI:** 10.1155/2013/273848

**Published:** 2013-05-30

**Authors:** Bruna Steiner, Valerio R. Aquino, Alessandra A. Paz, Lucia Mariano da Rocha Silla, Alexandre Zavascki, Luciano Z. Goldani

**Affiliations:** ^1^Infectious Diseases Section, Hospital de Clínicas de Porto Alegre, Universidade Federal do Rio Grande do Sul, Ramiro Barcelos 2350, Porto Alegre, RS 90035-903, Brazil; ^2^Hematology Section, Hospital de Clínicas de Porto Alegre, Universidade Federal do Rio Grande do Sul, Ramiro Barcelos 2350, Porto Alegre, RS 90035-903, Brazil

## Abstract

*Paecilomyces variotii* is a commonly occurring species in air and food, and it is also associated with many types of human infections. Pneumonia due to *Paecilomyces variotii* has been rarely reported in the medical literature. The authors report a 48-year-old patient with refractory lymphoma who underwent allogenic hematopoietic cell transplantation and developed pneumonia due to *Paecilomyces variotii*. They also review the published case reports of pneumonia caused by this fungus.

## 1. Introduction

*Paecilomyces* species are saprophytic fungi and are uncommon pathogens that can produce serious infections in immunocompromised patients and occasionally in immunocompetent hosts [1]. *Paecilomyces* is of clinical interest because of its pathogenicity and resistance to antifungal agents. *Paecilomyces variotii* is a commonly occurring species in air and food, and it is also associated with many types of human infections, such as fungemia, endocarditis, peritonitis, and osteomyelitis [2–9]. Pneumonia due to *Paecilomyces variotii* has been rarely reported in the medical literature.

Here, we report a patient with refractory lymphoma who underwent allogenic hematopoietic cell transplantation and developed pneumonia due to *Paecilomyces variotii*. They also review the case reports of pneumonia caused by this fungus.

## 2. Case Report

A 48-year-old Brazilian woman with the diagnosis of non-Hodgkin's lymphoma underwent chemotherapy and autologous hematopoietic cell transplantation in 2010. The lymphoma recurred, and the patient was admitted to the hospital to undergo chemotherapy and allogeneic hematopoietic cell transplantation in October 2011. She was conditioned with busulfan, melphalan, and gemcitabine and received peripheral blood stem cell (PBSC) from HLA sibling identical donor. Patient underwent prophylaxis of acute graft versus-host disease (aGVHD) with cyclosporine and methotrexate engraftment occurred on day 14 after transplant. During this period, she presented with febrile neutropenia and mucositis, which resolved after treatment with broad-spectrum antibiotics. On day 24 after transplant, the patient presented with probable pulmonary aspergillosis, as determined by an elevated serum galactomannan level. The patient was treated initially with intravenous micafungin (150 mg once per day) and was switched to oral voriconazole (200 mg twice per day). She also presented with cutaneous and intestinal Grade II aGVHD which improved after treatment with methyl prednisone 2 mg/kg. Voriconazole was suspended in December 2011 after 8 weeks of treatment. She was discharged on day 51 after transplant. 

 In February, she was admitted for the treatment of cytomegalovirus (CMV) esophagitis that was confirmed by an upper endoscopy showing esophageal ulcers and by histopathological examination showing cytomegalic inclusions. The patient was treated with intravenous ganciclovir (300 mg 12/12 hr) for 14 days with resolution of the ulcers.

**Figure 1:**
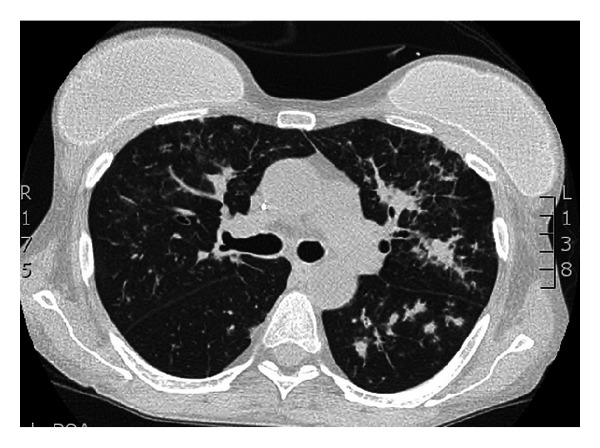
Chest CT scan showing confluent nodules and ground-glass patchy consolidations especially in the left lung.

The patient was readmitted in March 2012 with a respiratory infection and was treated with intravenous meropenem (1000 mg 8/8 hr) and vancomycin (1000 mg 12/12 hr). Because of the GVHD, the patient was maintained on chronic oral corticosteroids. She developed a productive cough, dyspnea, oliguria, and fever in April 2012. After blood, urine, and sputum samples were collected, the patient was started on piperacillin-tazobactam. Because tomographic images of the lungs showed nodules and ground-glass patchy consolidations (Figure 1) and because her serum galactomannan level was 2.95 ng/mL, the patient was started on oral voriconazole (200 mg 12/12 hr). She was also treated with linezolid due to clinical worsening and the growth of vancomycin-resistant enterococci (VRE) in the blood cultures. Because of her worsening ventilation, the patient was admitted to the intensive care unit (ICU) and was placed on nonmechanical invasive ventilation. At that time, her serum galactomannan level was 2.56 ng/mL. To optimize antifungal treatment for aspergillosis, intravenous micafungin (150 mg once per day) was combined with endovenous voriconazole. Due to respiratory failure that was not responsive to nonmechanical invasive ventilation, worsening of the hemodynamic status, and renal failure requiring hemodialysis, the patient was submitted to endotracheal intubation and a subsequent fiberoptic bronchoscopy. She was treated with linezolid for VRE for 15 days, and her blood cultures were subsequently negative. Direct microscopic examination of her bronchoalveolar lavage fluid revealed the presence of rare hyaline, dichotomous hyphae. Fungal cultures grew *Paecilomyces variotii*. The isolated fungus was identified based on its colony morphology and microscopic structures [14, 15]. The colonies were fast growing, flat, and cottony in texture. The mycelium was hyaline, septate, thin walled, and branched. Conidiogenous cells were branched, cylindrical, and loosely clustered, tapering gradually to a fine point. The conidia were globose and ellipsoidal and were produced endogenously to form a very long chain. Voriconazole was switched to liposomal amphotericin. No improvement was observed in the serum galactomannan level after 7, 14, and 21 days of treatment and remained stable (2.5 ng/mL).

 The patient presented with severe melena, and an endoscopy was performed, which revealed esophageal erosions and ulcers with gastric bleeding. The patient's hemodynamic parameters worsened significantly, requiring increasing doses of vasopressors, and her ventilatory patterns worsened. The patient died in May 2012.

## 3. Discussion

*Paecilomyces* is a cosmopolitan filamentous fungus that exists worldwide and inhabits the soil, decaying plants, and food products. This fungus closely resembles *Penicillium* fungi but can easily be differentiated by its loosely branched conidiophores and cylindrical conidiogenous cells with tapering tips. Some species of *Paecilomyces* have been isolated from insects. This fungus is usually considered a contaminant but may also cause infections in humans and animals. *Paecilomyces lilacinus* and *Paecilomyces variotii* are the two species most frequently associated with human disease. Other species reported to infect humans occasionally are *Paecilomyces marquandii* and *Paecilomyces javanicus* [14].

Pulmonary infections with pleural effusion and abscesses caused by *Paecilomyces lilacinus* have been described previously in two patients, one from Japan and one from Malta, with no apparent predisposing factors [16, 17]. We found that pneumonia due to *P*. *variotii* has only been previously reported in four patients (Table 1) [10–13]. Except for one patient, all patients including ours have been immunocompromised due to diabetes mellitus, hematological malignancies, or the use of chronic corticosteroids. The clinical presentations of the reported patients included fever, pleuritic chest pain, productive cough, and dyspnea. Chest imaging abnormalities included hilar lymphadenopathy, nodules, confluent and patchy opacities, and cavitary lesions. Despite appropriate antifungal treatment with amphotericin B, 2 out of 5 patients, including ours, had unfavorable outcomes. Recently, *Paecilomyces lilacinus* has been implicated as a cause of rhinitis and allergic alveolitis sustained by individuals living in substandard urban dwellings in proximity to decaying wood. We could not find any specific related exposure described in the reported cases of *Paecylomyces variotii* pneumonia [18].

In contrast to *Paecilomyces lilacinus*, which has shown poor susceptibility to amphotericin B, itraconazole, and echinocandins, *Paecilomyces variotii* has been shown to exhibit a different susceptibility pattern, being susceptible to most antifungal agents apart from voriconazole and ravuconazole [19–21]. Because of the technical difficulties frequently encountered with in vitro susceptibility testing for molds, the determination of the precise specie of *Paecilomyces* is important for the appropriate management and treatment of these infections. Recently, Houbraken et al. described a precise molecular method for the identification of *Paecilomyces* species based on the sequence of the intergenic transcribed spacer regions 1 and 2 (including the 5.8S rDNA) and a part of the beta-tubulin gene [22].

## 4. Conclusions

Pneumonia due to *P*. *variotii* is rare. Most of the patients are immunocompromised. The clinical manifestations are unspecific and include fever, pleuritic chest pain, productive cough, and dyspnea. Hilar lymphadenopathy, nodules, confluent and patchy opacities, and cavitary lesions are the most common imaging abnormalities. In contrast to *Paecilomyces lilacinus*, *Paecilomyces variotii* is susceptible to most antifungal agents including amphotericin B. Despite appropriate antifungal treatment with amphotericin B, patients can present unfavorable outcomes. Susceptibility tests are important for the appropriate management and treatment of these infections.

## Figures and Tables

**Table 1:** Case reports of *Paecilomyces variotii* pneumonia described in the medical literature.

Ref.	Place	Age/sex	Baseline disease	Diagnostic Sample	Therapy	Outcome
[10]	USA	67/male	None	Sputum	Itraconazole Posaconazole	Alive
[11]	USA	49/female	Pulmonary emphysema Use of chronic corticosteroids	BAL	AmB deoxycholate	Died
[12]	USA	33/male	DM-1	BAL	Ketoconazole AmB deoxycholate	Alive
[13]	UK	64/female	Hairy cell leukemia	BAL	AmB deoxycholate	Alive
Present case	Brazil	48/female	Non-Hodgkin's lymphoma	BAL	Lipossomal AmB	Died

Ref.: references; BAL: bronchoalveolar lavage; DM-1: diabetes mellitus; AmB: amphotericin B.
